# Epidemiology of Lyme Disease in a Highly Endemic European Zone

**DOI:** 10.3390/medicina56030115

**Published:** 2020-03-05

**Authors:** Agnė Petrulionienė, Daiva Radzišauskienė, Arvydas Ambrozaitis, Saulius Čaplinskas, Algimantas Paulauskas, Algirdas Venalis

**Affiliations:** 1 Clinic of Rheumatology, Orthopaedics Traumatology and Reconstructive Surgery, Institute of Clinical Medicine, Faculty of Medicine, Vilnius University, 01513 Vilnius, Lithuania; 2 Clinic of Infectious Diseases, Institute of Clinical Medicine, Faculty of Medicine, Vilnius University, 01513 Vilnius, Lithuania; daiva730jvg@gmail.com (D.R.); arvydas.ambrozaitis@elnet.lt (A.A.); 3 Centre for Communicable Diseases and AIDS, Vilnius 10105, Lithuania; saulius.caplinskas@ulac.lt; 4 Institute of Educational Sciences and Social Work, Mykolas Romeris University, 08303 Vilnius, Lithuania; 5 Department of Biology, Vytautas Magnus University, 44248 Kaunas, Lithuania; lgimantas.paulauskas@vdu.lt; 6 State Research Institute Centre for Innovative Medicine, 08410 Vilnius, Lithuania; algirdas.venalis@imcentras.lt

**Keywords:** Lyme, borreliosis, erythema migrans, epidemiology, Lithuania, Europe

## Abstract

*Background and objective:* Lyme disease, also known as Lyme borreliosis (LB), is a tick-borne infectious disease caused by the spirochete bacteria Borrelia. The risk of infection depends on the geographical area, ecological factors, and human behavior. Clinical manifestations of Lyme borreliosis have a wide range, but the most frequent clinical symptom, which is also a diagnostic symptom, is a skin rash called erythema migrans (EM). The disease is very common worldwide. In Lithuania, the disease frequency is 99.9 cases per 100,000 population (Centre for Communicable Diseases and AIDS, Lithuania, 2017). The main aim of this study was to obtain the baseline characteristics of the disease regarding the infected Lithuanian population. *Materials and Methods:* We analyzed data from the Centre for Communicable Diseases and AIDS about all Lyme disease (A69.2) diagnosed patients over a three-year period (from 2014 to 2016) in Lithuania. *Results:* In 2014–2016, 7424 (crude incidence rate 85.4) cases with LB were diagnosed in Lithuania. Most of them (4633 (62.4%)) were identified in women. Older people were more likely to suffer from LB. Urban residents were 2.6 times more often affected that those living in villages. Tick bites were primarily observed in high season months, from May to September (90%), with the highest peak in July. There was a higher number of observed tick bites (*p* = 0.003) in the urban residents. Erythema migrans occurred in 75.6% LB cases, while other symptoms did not exceed a quarter of all LB cases. There were 7353 (99.6%) cases where LB was confirmed via clinical symptoms and/or laboratory tests. Also, 1720 (23.2%) patients were tested for LB immunoglobulins. *Conclusions:* This study found a high incidence of Lyme disease in Lithuania. We elucidated the baseline characteristics regarding the infected Lithuanian population which may ease medical clinicians’ work on new Lyme diagnoses.

## 1. Introduction

Lyme borreliosis (LB) is the most common infection in Europe [1]. Global climate change has expanded the range of tick vectors, suggesting that LB will remain an important health issue in the forthcoming decades. It is possible to only approximate estimates of the LB incidence in Europe, because few countries report LB as a compulsorily notifiable disease. In Lithuania, this disease is mandatory notifiable. The calculated population-weighted average incidence rate for the regional burden of LB in Western Europe is 22.05 cases per 100,000 persons per year [2]. For example, in France, the annual incidence rate was 53/100,000 between 2009 and 2017 [3]. In Northern Italy (Lombardy), there were only 1.24 new cases per 1 million residents between 2000 and 2015 [4]. In the UK, the annual incidence is 12.1/100,000 [5], in Finland, it is 61/100,000 [6], and in Lithuania, it is 99.9 cases per 100,000 population (Centre for Communicable Diseases and AIDS, Lithuania, 2017), one of the highest rates on the continent. Spirochete bacteria is an etiologic Lyme disease agent that belongs to the Borrelia burgdorferi sensu lato group: B. burgdorferi sensu stricto (B.b.), B. garinii (B.g.), and B. afzelii (B.a.). Because of different Borrelia genospecies, clinical manifestations of Lyme disease have a wide range and differ by geographical region. B. burgdorferi sensu stricto (s.s.) is found in the Americas, and B. afzelii, B. garinii, and the less common B. burgdorferi s.s. are prevalent in Europe and Asia. Main clinical syndromes are as follows: neuroborreliosis (mainly in Europe (B. afzelii and B. garinii)), Lyme arthritis (mainly in the US (B. burgdorferi)), Lyme carditis, skin changes, erythema migrans, and borrelial lymphocytoma [7]. Erythema migrans is a diagnostic marker, and there is no need for additional laboratory tests. The diagnosis depends on clinical evaluation and is supported by laboratory tests. The enzyme-linked immunosorbent assay (ELISA) test is usually administered to detect Lyme disease immunoglobulins, followed by an immunoblot [8]. The risk of infection depends on the geographical area, ecological factors, and increase in human outdoor activities [9]. *Ixodes scapularis* (black-legged tick) is the most important tick responsible for spirochetes transmission to humans due to its wide distribution in many ecosystems [10,11,12]. The risk of human infection is greatest in the late spring and summer according to the tick’s life cycle. There are no vaccines against Lyme disease, only non-specific prophylaxis, such as wearing long and brightly colored clothes during outdoor activities, using insect repellents, avoiding tick-infected areas, and rapidly removing biting ticks [13,14].

The objective of this study was to elucidate the baseline characteristics regarding the infected Lithuanian population, which is important, because Lyme disease has a wide clinical manifestation range and must be managed by clinicians with different medical specialties.

## 2. Materials and Methods

All of the patients included in this study were diagnosed with Lyme disease (A69.2) in 2014–2016. The data were collected from the Centre for Communicable Diseases and AIDS of Lithuania. A retrospective material analysis was conducted. Some additional data were obtained from the website of the Centre for Communicable Diseases and AIDS of Lithuania (http://www.ulac.lt). Case rates are given per 100,000 population (the number of reported cases divided by the estimate of the population for that year multiplied by 100,000).

Frequency tables with percentages are presented for categorical data. Mean values (± SD) were calculated for quantitative data. Student’s *t*-test and chi-squared test were used to evaluate the differences between two independent quantitative and qualitative data sets, respectively. Crude rates were calculated for LB incidence in Lithuanian population. A two-tailed *p*-value less than 0.05 was considered to be significant. Statistical analysis was performed using R Commander (Rcmdr) version 2.3–2.

The study was approved by the Vilnius Regional Biomedical Research Ethics Committee (approval number 158200-17-900-420) and the State Data Protection Inspectorate.

## 3. Results

Between 2014 and 2016, 7424 LB cases were identified in Lithuania. Most of them (4633 (62.4%)) were identified in women. Older people were more likely to suffer from LB: 56.2% of LB cases were identified in people aged ≥ 51 years (Table 1). Women were statistically significantly older than men: mean (SD) age was 52.0 (± 361.59) and 44.3 (± 369.81) years for women and men, respectively (*p* < 0.001).

A total of 5368 (72.3%) infected people lived in urban territories, and the rest (2056 (27.7%)) were village residents. The number of LB-infected subjects living in the countryside statistically significantly increased and reached 857 (29.4%) in the last study years (*p* = 0.031) (Table 1).

The average crude LB incidence rate was 85.4 in 2014–2016, with the peak in 2016, when crude incidence rate was 101.6. Women remain the most infected with LB: the average crude LB incidence rate was 98.7 in women, meanwhile in men the crude incidence rate was only 69.7.

**Figure 1:**
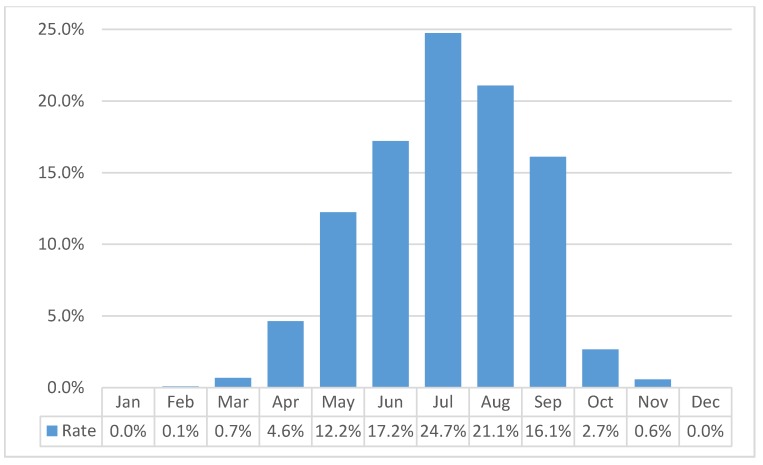
Tick bite LB infection frequency by months in 2014–2016.

Tick bites were observed in more than half of all LB cases—4576 (61.6%). There was no statistically significant difference for tick bites ratio between men and women: 62.8% and 60.9%, respectively (*p* = 0.107). Frequency rate of clinical symptoms was the same between groups of LB patents with and without tick bites (for all symptoms, *p* < 0.05). Tick bites were primarily observed in high season months, from May to September (90%), with the highest peak in July (Figure 1). There was a higher number of tick bites (*p* = 0.003) in the urban residents.

**Figure 2:**
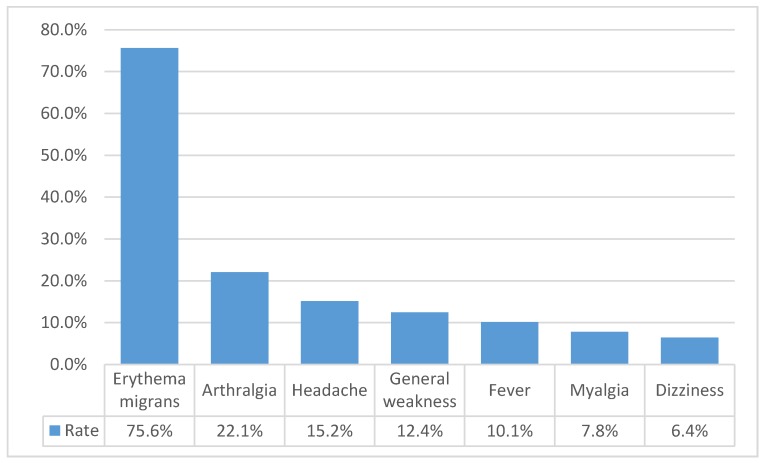
The most frequently observed symptoms of Lyme disease in 2014–2016.

Only 996 patients had complete data regarding their clinical symptoms, and for the remaining 6428, the data were unknown. Erythema migrans occurred in 753 (75.6%) LB cases and remained the most frequent symptom, while other symptoms were observed in less than a quarter of all LB cases (Figure 2). Clinical symptoms were independently distributed between men and women (*p* = 0.651), but subjects with clinical symptoms were clinically significantly older: mean age 52.1 (± 18.30) vs. 48.7 (± 19.59) years (*p* < 0.001). Urban people suffered from erythema migrans more frequently than village residents (*p* < 0.001).

Determination of clinical symptoms was the most frequent method for LB confirmation with 5357 (72.2%) cases. Clinical symptoms were evaluated with laboratory tests, and LB was confirmed in additional 1849 (24.9%) cases. Finally, only 189 (2.5%) cases were confirmed only via laboratory tests. In total, there were 7353 (99.6%) cases where LB was confirmed via clinical symptoms and/or laboratory tests. Furthermore, 1720 (23.2%) patients were tested for LB immunoglobulins: 1283 (17.3%) were IgM-positive IgM, 952 (12.8%)—IgG-positive, and 536 (7.2%) were both IgM- and IgG-positive. Interestingly, village residents were more frequently tested for LB immunoglobulins (26.0% vs. 22.1% (*p* < 0.001)).

Among those who reported erythema migrans, 277 (36.8%) were tested for B. burgdorferi immunoglobulins, 198 (71.5%) were IgM-positive, 142 (51.3%)—IgG-positive, 67 (24.2%) were both IgM- and IgG-positive, 3 (1.1%)—both IgM- and IgG-negative.

## 4. Discussion

As Lyme disease is very common throughout Europe, except in the coldest areas in the north (e.g., the crude incidence rate in Iceland is 2.0 cases per 100,000) [15], and the hottest areas in the south (e.g., the crude incidence rate in Spain is 2.5–11.6 cases per 100,000), [16], not surprisingly, Lithuania is a highly endemic Lyme disease country. The disease’s incidence range is 73.9–100.6 cases per 100,000 population in the past eight years (2011–2018). To compare, it is similar to other high disease incidence European countries: parts of Slovenia, Germany, and Austria, the Baltic coastline of southern Sweden, and some Estonian and Finnish islands, where more than 100 cases per 100,000 population are observed [17].

Due to the global warming and milder winters leading to a longer tick activity period, probably, the number of new disease cases will remain high or become even higher in the future. A higher number of infection cases may even be caused by better disease understanding among both healthcare professionals and patients.

Women are affected more frequently than men. Gender distribution is the same as reported in other European countries: Finland [6], Norway [18], Sweden [19], and Germany [20], with the exception of England and Wales, where men are prevalent [21]. This may be because women pay more attention to their skin changes and turn to healthcare when they are in the erythema migrans stage of the disease.

Lyme disease primarily affects those in middle age (51–60 years of age) and adult urban residents, i.e., it occurs one decade earlier than according to other authors’ records [6,21]. We did not find the bimodal age peak of onset, which is first in early childhood, then in age group of 60–70 years, and is how it is usually reported, and the reason remains unclear, it might be because pediatricians are less likely to report than others [6,20,21]. Age distribution of the other, also endemic, tick-borne disease—tick-borne encephalitis is very similar to Lyme disease in Lithuania, and it occurs mostly in middle age (45–54 years) [22].

The most frequently observed symptom is erythema migrans, which is also a diagnostic marker of the disease. If not treated in the erythema migrans stage, Lyme disease can lead to some serious health problems: neuroborreliosis, Lyme arthritis, atrophic chronic acrodermatitis, can cause an atrioventricular Block. Over one-third of patients with documented skin rash—erythema migrans (EM) were tested for Lyme disease immunoglobulins, mostly in villages. The higher reported incidence of additional laboratory tests in villages may be explained by the lack of knowledge of the disease among medical doctors. According to the latest European and American guidelines for the diagnosis of Lyme borreliosis, no additional Lyme disease laboratory tests are necessary in cases of erythema migrans [8,23]. Thus, it may be over-diagnosed. When there is no evidence of erythema migrans, a combination of clinical presentation and laboratory testing has to be used.

The peak of observed tick bites is similar to the peak of ticks, which occurs in the warmest season of the year, between May and September, with a higher incidence in July. The peak of observed tick bites also depends on human activities, e.g., higher outdoor activity during the warmest season of the year. *Ixodes ricinus* (*I. ricinus*) ticks are responsible for transmitting Borrelia bacteria to humans through a bite. Temperature and relative humidity are key requirements for the development, survival, and activity of *I. ricinus* [24]. In Europe, B. afzelii and B. garinii predominate, different Borrelia genospecies cause different clinical syndromes of the disease. There is still lack of information on the type of Borrelia ticks are infected with in Lithuania; furthermore, of the amount of ticks infected with Borrelia bacteria is unknown. This requires further research.

We acknowledge some limitations of this study. Not all diagnosed Lyme borreliosis cases are included on the national Communicable diseases register, because not all healthcare professionals provide such information, especially the ones who work in private care units. What is more, the data we have analyzed does not give any information about the most frequent clinical syndromes of the disease: Erythema migrans, Neuroborreliosis, Lyme arthritis, Lyme carditis.

## 5. Conclusions

The article highlighted the high frequency of Lyme disease in Lithuania, which is why LB remains an important public health concern. We elucidated the baseline epidemiological characteristics regarding the infected Lithuanian population, which is important, because Lyme disease has a wide clinical manifestation range and must be managed by clinicians from different medical specialties. Most often, the disease affected female urban residents over 50 years of age; the higher risk of infection is the warmest time of the year, between May and September, especially in July. The most often observed clinical symptom was skin rash—erythema migrans. While there are no vaccines available, public education and better maintenance of public areas might help to prevent the disease; furthermore, it is very important to diagnose and to treat the disease as early as possible to prevent more serious health problems.

## Figures and Tables

**Table 1:** Demographics of Lyme borreliosis-infected subjects in Lithuanian 2014–2016 period.

Demographics	Calendar Years			Total *n* (%)
	2014, *n* (%)	2015, *n* (%)	2016, *n* (%)	
Total	2257 (30.4)	2252 (30.3)	2915 (39.3)	7424 (100.0)
Age, years				
Mean (± SD)	48.5 (19.61)	48.9 (19.57)	49.8 (19.23)	49.1 (19.45)
Range	1–90	1–90	0–91	0–91
0–10	112 (5.0)	116 (5.2)	124 (4.3)	352 (4.7)
11–20	118 (5.2)	127 (5.6)	154 (5.3)	399 (5.4)
21–30	225 (10.0)	183 (8.1)	232 (8.0)	640 (8.6)
31–40	248 (11.0)	267 (11.9)	353 (12.1)	868 (11.7)
41–50	374 (16.6)	357 (15.9)	461 (15.8)	1192 (16.1)
51–60	479 (21.2)	502 (22.3)	657 (22.5)	1638 (22.1)
61–70	404 (17.9)	411 (18.3)	520 (17.8)	1335 (18.0)
71–80	249 (11.0)	250 (11.1)	352 (12.1)	851 (11.5)
≥81	48 (2.1)	39 (1.7)	62 (2.1)	149 (2.0)
Gender				
Women	1430 (63.4)	1427 (63.4)	1776 (60.9)	4633 (62.4)
Men	827 (36.6)	825 (36.6)	1139 (39.1)	2791 (37.6)
Residence				
Village	600 (26.6)	599 (26.6)	857 (29.4)	2056 (27.7)
Urban	1657 (73.4)	1653 (73.4)	2058 (70.6)	5368 (72.3)

SD: standard deviation.
